# A systematic review of strategies in digital technologies for motivating adherence to chronic illness self-care

**DOI:** 10.1038/s44401-025-00017-4

**Published:** 2025-04-26

**Authors:** Tianqin Lu, Qingyuan Lin, Bin Yu, Jun Hu

**Affiliations:** 1 Department of Industrial Design, Eindhoven University of Technology, Eindhoven, The Netherlands; 2 Digital Life Centre, Amsterdam University of Applied Sciences, Amsterdam, The Netherlands

**Keywords:** Health care, Information systems and information technology

## Abstract

The global incidence of chronic diseases is rising, posing substantial social and economic challenges. These conditions necessitate effective long-term self-care, which can be supported by digital interventions using remote measurement technologies, like smartphones and wearables. This systematic review investigates the motivational strategies within digital technologies to improve self-care adherence for chronic illnesses, particularly cardiovascular diseases and diabetes mellitus. A literature search was conducted, focusing on studies from 2004 to 2024. A total of 17 studies met the inclusion criteria. The reviewed interventions targeted medication adherence, lifestyle modifications, and symptom tracking. Findings suggest that motivational strategies, such as feedback, health literacy, reminders, and motivational messages, goal-setting, social interaction, gamification, and rewards can improve patient adherence to self-care behaviors. However, their effectiveness relies on theoretical grounding, data-driven features, and personalization. Future research should prioritize integrating robust theories and developing standardized metrics for adherence to enhance the reliability and impact of digital interventions.

## Introduction

According to^1^, the incidence of chronic diseases is increasing globally every year, leading to severe social and economic consequences^2^. Chronic diseases normally persist over an extended period and progress slowly, affecting individuals’ quality of life and necessitating long-term monitoring and management^3,4^. Cardiovascular diseases (CVDs) and diabetes mellitus (DM) are two of the most common chronic diseases due to their high prevalence, morbidity, and mortality rates^5^. CVDs refer to heart and blood vessel disorders, which are chronic physical diseases that evolve gradually over time^6^, which can lead to heart failure, stroke, and sudden death^7^. DM is a metabolic disorder characterized by abnormally high blood sugar levels^8^. It damages many body systems, particularly blood vessels, eyes, kidneys, heart, and nerves^9^. DM is also well-recognized as a main risk factor for the development of CVDs^10^. Moreover, patients with a combination of DM and cardiovascular diseases reported nearly double mortality rates and reduced life expectancy by ~12 years^11^.

Self-care is vital for both the prevention and treatment of chronic diseases^12^. Effective self-care enables patients to maintain independent lifestyles, improving quality of life, reducing hospitalizations, and enhancing clinical outcomes^13^. According to the middle range theory of self-care of chronic Illness, self-care is defined as “a process of maintaining health through health promoting practices and managing illness," which encompasses three key elements: maintenance, monitoring, and management^14^. Maintenance refers to adherence to behaviors for maintaining physical and emotional stability; monitoring means observing changes in signs and symptoms; management requires responding to these changes^13,14^.

Remote measurement technologies (RMTs), such as smartphones, wearables, and associated applications, have been extensively studied to deliver digital interventions to support self-care among patients with chronic diseases^15,16^. These information-gathering technologies enable continuous monitoring outside clinical settings, providing patients with data and feedback and facilitating a more personalized and effective approach to health management^17,18^. RMTs refer to digital technologies that are able to remotely gather various types of data about human behavior, psychological states, and physiological parameters^16,19^. RMTs commonly use two approaches to data collection: active and passive. Active RMTs require users to actively input data, which include subjective measures like mood and pain, and behavioral data, such as medication adherence and alcohol consumption^20–22^. Passive RMTs automatically collect information from users through device-embedded sensors, tracking metrics, such as heart rate, skin conductance, step count, and activity levels^23,24^.

Successful RMT-based digital interventions require patients to adhere to active data collection, which requires user effort and accurate self-reporting^22^. While RMTs can offer valuable feedback and support in acquiring health behavior information and skills, patients often face challenges in maintaining motivation over time to use RMTs^25,26^. According to the information-motivation-behavioral (IMB) model^27^, motivation (personal and social) is a key determinant of adherence behavior^28^. The declining motivation can directly reduce patients’ adherence to RMTs, decreasing the effectiveness of overall self-care outcomes^26,29^. Therefore, understanding and addressing motivational factors is important for enhancing the efficacy of RMTs and supporting sustained self-care.

Furthermore, how patients interact with and interpret health data collected from RMTs can significantly impact the patients’ motivation to use RMTs and their adherence to self-care^30^. Data is not merely a fact but requires thoughtful engagement to be meaningful^31^. Recent studies have focused on facilitating patients’ understanding and utilization of their health data through various methods, such as data visualization^30^ and AI-driven chatbots^32^. Beyond these methods, the design of data presentation in RMTs can also impact motivation. For example, in study^33^, virtual crowns and trophies were used as rewards to recognize users when they achieved specific goals aimed at boosting motivation. However, it remains unclear which motivational strategies are employed and how these strategies affect long-term adherence to self-care.

Therefore, the objective of this systematic review is to assess the current breadth of evidence on the use of motivational strategies within digital intervention to promote self-care adherence among patients with CVD or DM. This review specifically aims to (1) examine the types of RMTs used, (2) identify the motivational strategies and associated theories employed, (3) inventory the design of data presentation implemented in these RMTs, and (4) assess how adherence outcomes were evaluated and reported. To our knowledge, the only review that has explored motivational strategies to increase participation and adherence in cardiac rehabilitation was recently published by ref. ^34^. However, this work did not examine self-care adherence but rather focused on content like motivational interviewing and counseling delivered by healthcare professionals^34^. Another study^35^ on quantifying the quantified self-focused on understanding patients’ motivations for self-tracking health data. However, it did not identify specific motivational strategies that could be integrated into health interventions^35^. Moreover, previous studies on adherence have generally focused on RMTs addressing specific behaviors or activities within self-care. For instance, one study evaluated the effectiveness of app-based interventions in improving medication adherence in CVDs^36^. Another review focused exclusively on wearable activity tracker-based interventions aimed at improving adherence to physical activity and health outcomes in chronic disease contexts^37^. Considering these limitations in the scope of previous reviews, the increasing amount of research on RMTs, and the importance of motivation for sustained adherence to self-care practices, there is a need for an up-to-date and comprehensive systematic review.

This systematic review summarizes the research on motivational strategies in digital interventions from 2004 to 2024. A comprehensive search across four electronic databases yielded 17 studies for inclusion. The included studies are analyzed from the perspective of the types of RMTs, the motivational strategies and theories applied, the data presentation designs, and the evaluation methods for patient adherence. This review reveals that while various strategies and designs have been employed to motivate patient adherence to self-care, challenges remain in the research and development of digital interventions. Insights from this review contribute to the growing field of health information technology, particularly in how digital tools like RMTs can enhance chronic disease management. By integrating motivational strategies into self-management systems, these technologies can improve healthcare delivery. This knowledge may inform the design of health information technologies, such as remote monitoring tools and self-management platforms, to improve patient engagement, adherence, and health outcomes. This review also highlights areas for improvement, emphasizing the need to address existing gaps to develop more effective and sustainable digital interventions for chronic disease self-care.

## Results

This section presents the findings derived from the comprehensive analysis of the included studies, structured based on the data examined for each research question listed in “Research questions”.

### Search results

**Fig. 1:**
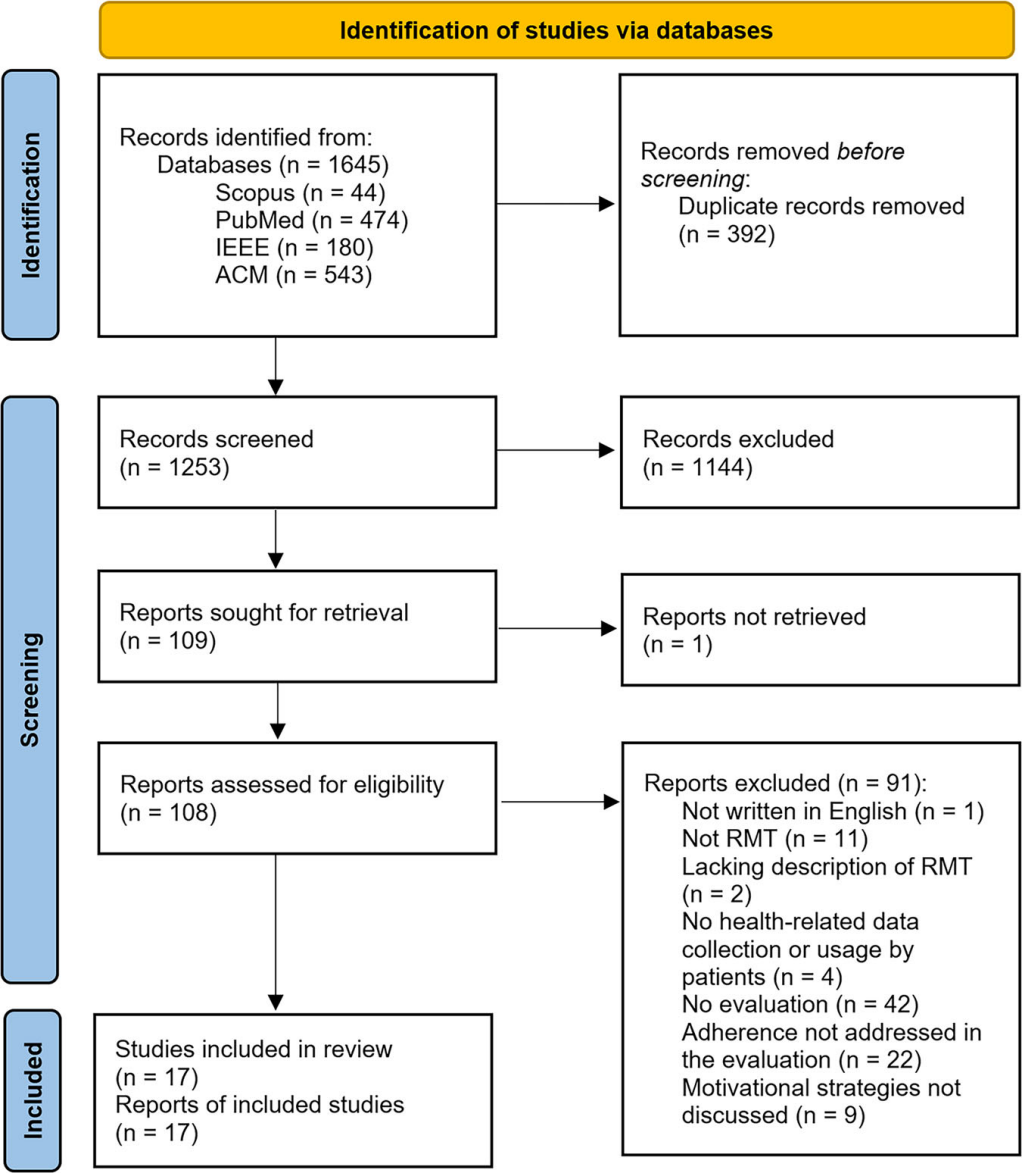
PRISMA flow diagram of included and excluded studies. The PRISMA flow diagram illustrates the study selection process for this systematic review. Records retrieved through searches were assessed for relevance, with exclusions and reasons documented. Studies meeting the criteria were included in the final analysis.

The initial search across four electronic databases returned a total of 1645 articles, of which 324 were identified as duplicates. The titles and abstracts of the remaining 1321 articles were screened, resulting in the exclusion of 1114 articles. As a result, 109 papers proceeded to full-text review. However, one paper could not be obtained in full text and was therefore excluded from the review. Based on the eligibility criteria, 17 studies were selected for detailed review and data extraction. The full screening process and the reasons for exclusion are summarized in Fig. 1.

### Study characteristics

The 17 studies included in this review were published between 2011 and 2023, focusing on patients with diabetes and/or various CVDs. Geographically, 11 studies were conducted in the United States, with the remaining studies conducted in China (*n* = 1), South Korea (*n* = 1), Japan (*n* = 1), and Spain (*n* = 1). Additionally, one study was conducted across three locations: Spain, Italy, and the Czech Republic. These studies analyzed various types of RMTs and their associated digital interventions, employing diverse study designs, including six randomized controlled trials (RCTs), four pre-post studies, two longitudinal studies, two feasibility studies, one crossover study, and one formative in-situ evaluation study, with duration ranging from two weeks to 12 months. Sample sizes varied considerably, ranging from 10^38^ to 450^39^. 11 of the 17 studies (~65%) involved fewer than 50 participants, reflecting smaller-scale trials. The participant populations across these studies predominantly consisted of older adults. Detailed characteristics of each included study can be found in Table 1.

**Table 1:** Characteristics of the included studies

Study	Country/Site	Target self-care behaviors or purposes	Description of RMT device	Type of RMT	Description of interventions	Experimental design and duration	Participants	Adherence measurement	Adherence outcome
^40^	United States	To improve self-care behaviors and medication adherence	A smartphone application, HeartMapp	Active Passive	HeartMapp + BioHarness-3 chest strap: daily weighing, symptom assessment, responding to tailored alerts, vital sign monitoring; feedback; alerts; medication tracker; education; graphical displays	2-arm RCT (Intervention group - HeartMapp vs. Control group - HF education only), 4-week	*N* = 18 (Ni = 9, Nc = 9), Age = 53.06 HF	Application usage: the duration for which the participants accessed HeartMapp features. Medication adherence: 8-item self-administered Morisky Medication Adherence Questionnaire	Participants used HeartMapp features 78% of the time on average, with daily access at 43%. Medication adherence improved in both groups, but not significantly (*P*=0.53), with the HeartMapp group showing a mean change of 0.23
^43^	United States	To improve SMBG	A mobile intervention system that combined a glucometer with a data transmitter and cell phone	Passive	Mobile ICT System: Real-time BG monitoring; social (peer) support via automated text feedback	Non-randomized feasibility study, 3-month	*N* = 15 Age = 49.3 Diabetes	BG monitoring: the frequency of SMBG transmissions	73% of participants transmitted BG readings within 5 days. 20 to 30% of participants did not adhere well to the SMBG schedule
^41^	United States	To improve medication adherence and BP monitoring	An mHealth medication and BP self-management system (SMASH) that combined a smartphone application for BP data transmission and a Bluetooth BP device for daily monitoring	Passive	SMASH: BP monitoring; medication reminders through MedMinder tray; real-time feedback; alerts and motivational messages	2-arm RCT (Intervention group: SMASH vs. Control group: SOC), 3-month	*N* = 19 (Ni = 6, Nc = 10), Age = NR Hypertension	Medication adherence: the timing of medication intake relative to the prescribed dosing time	The intervention group had significantly higher adherence rates compared to the standard care control group
^49^	Spain, Italy, Czech Republic	To improve therapy adherence (drug intake, food intake, and PA prescriptions) and enhance patients’ willpower in managing their disease	A patient mobile device and a professional control panel: the METABO system	Active Passive	METABO System: monitoring BG, diet, PA, and medication adherence; personalized feedback through automated messages; education and quiz	2-arm RCT (Intervention group - METABO vs. Control group - SOC), 4-week	*N* = 51 (Ni = 26, Nc = 14), Age=NR Diabetes	The degree to which a patient follows medical prescriptions: Food intake adherence: percentage of ingested kilocalories compared to the recommended daily calorie intake; glycaemic self-check inputs adherence: percentage of hypo/hyperglycemic episodes compared to the expected number of unstable glycemic events; PA adherence: ratio of total weekly metabolic equivalents of task to the prescribed physical activity duration; educational content adherence: total score from completed questionnaires divided by the number of prescribed quizzes	The results indicated an overall improvement in adherence across all categories
^44^	Spain	To improve medication adherence and healthy lifestyle management	A web and smartphone-based medication self-management platform, Medplan	Active	Medplan: monitoring medication adherence; medication reminders; healthy lifestyle advice; clinician communication	Single-arm PPS, 6-month	*N* = 42 Age = NR Hypertension, dyslipidemia, HF, human immunodeficiency virus infection	Medication adherence: the proportion of days covered with medication (PDC) and Simplified medication adherence questionnaire (SMAQ)	No differences were found in adherence to medication measured by proportion of days covered with medication (PDC); adherence significantly improved when measured using SMAQ, with a reduction in the number of days with missed doses
^45^	United States	To improve BP monitoring, weight management, diet, PA, and smoking cessation	An interactive Web-based system integrated with the electronic health record (EHR): the EMPOWER system	Active	EMPOWER + wireless BP monitor + pedometer + dashboard: Monitoring BP, steps; goal setting and tracking; automated feedback; alerts; EHR integration; graphical displays; education; communication with care team	Single-arm PPS, 6-month	*N* = 147 Age = 62.2 Uncontrolled BP	Both a 14-day average of home BP data and frequency of BP uploading	NR
^38^	United States	To adjust immediate meal options, and plan future meals	A smartphone application, GlucOracle	Active	GlucOracle: meal logging; nutritional assessment; feedback; personalized prediction; graphical displays	Feasibility study (Experienced Adopters vs. Novice Adopters), 2–5 weeks	*N* = 10 (Ne = 5 Age = 54, Nn = 5, Age = 55) Diabetes	Participants’ usage logs for frequency of use for different features overall and over time	Meal logging declined over time, from 15 (experienced) and 8 (novices) meals/week initially to 7 and 6 meals/week by week 4
Study	Country/Site	Target self-care behaviors or purposes	Description of RMT device	Type of RMT	Description of interventions	Experimental design and duration	Participants	Adherence measurement	Adherence outcome
^46^	China	To improve patient compliance with hypertension self-management	A mobile application, BP Assistant (BPA)	Active	Application: monitoring, medication, diet, exercise, symptoms; instant feedback; alerts, reminders, reports, and competitive leaderboards; education	4-Arm comparative study (Version 1: Management plan, reminder service, health check-up vs. Version 2: Version 1 adds health education vs. Version 3: Version 2 adds health report vs. Version 4: Version 3 adds leaderboard), 2-month	*N* = 143 (Nv1 = 36, Nv2 = 39, Nv3 = 39, Nv4 = 32) Hypertension	The frequency of BP measurements	Patient compliance improved as functional modules were added to the application and was maintained at a high level (0.73). Total compliance increased from 0.54 to 0.73, a significant difference (*P*<0.001)
^47^	United States	To promote self-monitoring of symptoms, enhance health literacy, and boost confidence in managing health	A mobile application, Healthy Heart	Active	Healthy Heart: monitoring weight and symptoms; customized feedback; graphical displays; education; alerts	Single-arm PPS, 4-week	*N* = 12 Age = 57.83 Heart failure	Self-care maintenance, management were measured used the Self-Care of Heart Failure Index (SCHFI) Version 6	The maintenance, management scales showed clinically relevant improvement from baseline to post-test as the difference in the mean baseline score for maintenance and management was greater than 8
^52^	United States	To improve self-efficacy and health-related behaviors	A mobile application, capABILITY	Active	capABILITY: monitoring carbohydrate consumption, BG, exercise; behavioral trigger messages (Sparks: designed for individuals who could benefit from motivational support; Facilitators: designed for individuals who lack ability); education; goal tracking and setting	3-crossover factor study (capABILITY with Facilitator trigger group, capABILITY with Spark trigger group and Control group: capABILITY without triggers), 9-week	*N* = 20 Age = 54.7 Diabetes	Behavioral task adherence measured by total time in capABILITY	Participants within the spark group produced a 75.1% (133/177) behavioral task adherence which was the highest among the three groups (not statistically significant)
^39^	United Kingdom	To prompt the use of skills such as self-monitoring and goal-setting and to empower patients to increase their levels of PA long term	A health’ web-based program, e-coachER	Active Passive	e-coachER + pedometer + fridge magnet: monitoring daily steps or minutes of moderate and vigorous PA; goal setting and viewing; feedback; personalized e-mail/text reminders, motivational messages	2-arm RCT (Intervention group: exercise referral schemes plus e-coachER vs. Control group: exercise referral schemes only), 12-month	*N* = 450 (Ni = 224 Age = 50, Nc = 226 Age = 21) Weight loss, low mood, osteoarthritis, diabetes and high blood pressure	Adherence to PA using a composite measure to describe the proportion of participants in each arm of the trial who achieved at least 150 minutes of moderate and vigorous PA in bouts of at least 10 minutes at 4 months and were still doing so at 12 months	PA primary outcomes at 12 months were only small and not significant
^51^	United States	To improve upper limb motor deficits rehabilitation	A smartphone application, RehabPhone	Passive	RehabPhone (with built-in accelerometer and gyroscope sensor): monitoring the user movement in the x, y, and z dimensions, detecting duration and repetitions and smoothness of activities; feedback; graphical displays	Single-arm LS, 6-week	*N* = 12 Age = 62 Stroke	Adherence to therapies: the degree to which the person’s behavior corresponds with the agreed recommendations from a health care provider	User adherence is over 90%
^33^	United States	To manage multiple health goals in collaboration with care managers	A tablet application	Active	Application: goal setting and tracking; awards (virtual crowns and trophies); reminders; communication with care manager	Formative evaluation study, 24-week	*N* = 20 Age = 65.7 Mild to no cognitive disabilities (include Hypertension, HF)	Frequency and consistency of goal tracking, usage logs of the application	Participants reported increased commitment to goals and adoption of healthy behaviors
^48^	South Korea	To aid the SMBG, diet planning, and PA	A mobile application, iCareD	Active Passive	iCare + glucometer: SMBG, dietary habits, and step count; automated text messages (educational, behavioral, and motivational messages); EMR integration; personalized recommendations and bidirectional feedback; goal setting; graphical displays; clinician communication	3-arm RCT (Group 1: usual care vs. Group 2: mobile diabetes self-care (MC) vs. Group 3: MC with personalized, bidirectional feedback from physicians), 26-week	*N* =269 Age = 52.5 (N1 = 87, N2 = 91, N3 = 91) Diabetes	Physical activity’s goal achievement rate (daily step counts), the frequency of SMBG, and documenting diet records	The participants were comfortable with using the iCareD system and exhibited high adherence
^50^	United States	To motivate the exercise of the paretic upper extremity	A moblie application, Yousician	Passive	Yousician + a piano keyboard with built-in speakers: capture the music that the user plays; real-time auditory feedback; concurrent visual feedback on timing accuracy and key-pressing correctness; rewards (starts and “certification")	Single-arm PPS, 3-week	*N* = 10 Age = 56.79 Stroke	Application usage: an individual’s usage (minutes) of the application	No trend of declined usage during the 3 weeks; some participants remained low usage throughout the study
^42^	Japan	To improve self-monitoring of vital signs and health conditions	A mobile application and a web-based dashboard, Mimamori-cho	Active	Mimamori-cho: monitoring BP, heart rate, body weight, percent fat, body temperature, oxygen saturation, symptoms/signs, sleep, PA, daily salt intake, dietary, exercise records, and medication records; automated alerts; educations; motivational messages	2-arm RCT (Intervention group: Mimamori-cho vs. Control group: using the HF diary), 2-month	*N* = 24 Age = 60 (Ni = 13 Age = 55, Nc = 11 Age = 60) Heart failure	Adherence to self-monitoring of four vital signs (BP, body weight, body temperature, and oxygen saturation) was based on patient records in the application (intervention group) or HF diary (control group). Adherence was defined as recording each vital sign at least once daily, with the adherence ratio (%) calculated as the number of days self-monitored divided by the total study duration.	No significant differences in adherence to self-monitoring of vital signs between the intervention and control groups, with both groups achieving high median adherence rates of around 100%
^53^	United States	NA	A mobile application, HyperCoach	Active	SMBP plus weight kit (a weight scale and a BP cuff device and mHealth coaching): monitoring weight and BP; goal setting; automated reminders; graphical displays; education; self-assessment quizzes	Single-arm LS, 60-day	*N* = 34 Age = 44.8 Hypertension	Total time spent on educational material, total educational content completed, BP and weight readings taken, and assessments and quizzes completed	Participant adherence increased from an average of 26.5 out of 30 measurements (SD=4.47) during the health awareness phase to 28.74 out of 30 (SD=1.92) during the health coaching phase

*BG* blood glucose, *SMBG* self-monitoring blood glucose, *BP* blood pressure, *HR* heart rate, *HF* heart failure, *PA* physical activity, *RCT* randomized controlled trial, *N/A* not applicable, *NR* not reported, *NI* number of participants in the intervention group, *NC* number of participants in the control group, *SOC* standard of care, *EHR* electronic health records, *EMR* electronic medical records

Table 1 outlines the characteristics of the included studies in this review. It is structured into ten columns: study, country/site, target self-care behaviors or proposes, description of the RMT devices, type of RMT (active or passive), study design and duration, participants, adherence measurement methods, and adherence outcomes.

### Digital interventions

Thirteen interventions in the included studies had multiple aims, primarily focusing on improving adherence to self-monitoring and management, such as medication intake and the tracking of symptoms and vital signs, like blood pressure and blood glucose levels^39–48^, to notice and focus on specific health problems. Six studies also emphasized adherence to lifestyle, including weight management, physical activity, dietary changes, and smoking cessation^38,44–46,48,49^. Two studies focused on the rehabilitation for motor deficits and the promotion of physical activity^50,51^. Additionally, many studies sought to empower patients by improving their self-efficacy^33,41,45,47,52–54^ and health literacy, as seen in^42,45,47,53^, educating patients about the fundamental disease knowledge, the risks associated with unhealthy behaviors, and the benefits of adopting healthy behaviors.

Interventions in the included studies take different forms and involve healthcare professionals on different levels. User control refers to interventions where patients have a primary role in managing their own care through digital interventions, including making decisions based on the data they collect and use. Expert control emphasizes the primary role of the healthcare professional, where they supervise the intervention and provide guidance to patients about their behavior. Among the interventions reviewed, 11 were based on user control^38–43,46,47,50,51,53^. These interventions allowed patients to manage their health independently, with minimal direct involvement from healthcare providers.

Five interventions incorporated both user and expert control^33,44,45,49,52^, where patients engaged in self-care activities while healthcare professionals monitored the tracked data and sent motivational or reminder messages at specific times. For example^44^, provided patients with control over daily medication management and adherence tracking, while healthcare professionals set up the medication plan and maintained communication with patients that facilitated ongoing support. Similarly, in^45^, patients were responsible for daily monitoring, goal setting, and accessing educational content, but healthcare professionals played a crucial role in guiding treatment, monitoring patient data, and adjusting care plans as needed. Additionally, one study^48^ employed two intervention groups: one with only user control, where patients input their self-care data (e.g., blood glucose, dietary habits, and step count), and another with both user and expert control, featuring bidirectional feedback from physicians.

The health-related metrics monitored in the RMT-based interventions include blood glucose levels, blood pressure, weight, symptoms, diet, physical activity, and medication adherence. 75% of interventions (*n* = 12) extended beyond mere data tracking by incorporating feedback mechanisms, ranging from real-time feedback to personalized and behavioral trigger messages. Additionally, nine interventions provided tailored educational resources, and one offered health reports to enhance user knowledge and support health management. Goal-setting and tracking features were included in six interventions. Moreover, reminders and alerts were integrated into six interventions each to support adherence and engagement. Social support, involving peer or family participation, was featured in two studies. Some of these also served as motivational strategies, which are further elaborated in Section "Motivational strategies to improve patient adherence"

This review identified 14 interventions that are based on mobile applications. Of these, four were smartphone-based^38,40,41,51^, one was implemented via a tablet^33^, and the remainder did not specify the type of device^42,43,46–50,52,53^. Additionally, two interventions were web-based^39,45^, while one was accessible through both web and smartphone interfaces^44^.

RMTs varied in their application across the studies. Four studies employed passive RMTs, such as pedometers, built-in accelerometers, and gyroscope sensors, to unobtrusively monitor physical activity without requiring user input from participants^41,43,50,51^. The majority of the studies (*n* = 9) utilized active RMTs, which necessitated higher levels of user interaction. These included mobile systems for time-stamped electronic logs, patient-reported physical activity outcomes, dietary records, and wireless monitoring devices, such as blood pressure monitors and weight scales^33,38,42,44–47,52,53^. Furthermore, four studies incorporated both active and passive RMTs, combining automated data collection with participant-reported metrics^39,40,48,49^.

Data plays a crucial role in the design and functionality of the digital interventions reviewed in this study. Across various interventions, the collected data from patients was used not only to record and monitor patient progress but also to personalize feedback, tailor messages, and assist care providers in decision-making. Eight studies specifically reported the use of graphical displays within the RMTs; details can be found in Table 2. Of these, three studies employed line graphs^47,51,53^, two used color-coded indicators^47,48^, and one utilized a horizontal bar display^38^ and two studies mentioned the inclusion of data visualizations^33,45^. These visualizations were intended to help patients track their progress over time and aid understanding of their health data. However, the limited descriptions of these graphical elements across the studies restrict our ability to fully assess their design and effectiveness in driving patient engagement.

**Table 2:** Health data and visualization

Study	Data	Visualization / Role of data
^40^	Weight, blood pressure, heart failure symptoms, and physiological measures	A graphical module that displays trends in patient performance
	Weight and heart failure symptoms classification	Color-coded indicators four zones (green, yellow, orange, red)
^45^	The patient-generated home data	Not reported
^38^	Macronutrient of the meal, e.g., carbs, protein	Horizontal bar
^47^	Weight	Line graph
^51^	Repetitions of the “Horizontal bowl" activity	Line graph
^48^	Glucose levels	Color-coded indicators
^33^	Progress	Dynamic visualizations to demonstrate progress which were designed in consultation with the study participants
^53^	Blood pressure and weight trends as well as daily and weekly averages	Line graph

Table 2 summarizes the types of health data and their corresponding visualizations or roles in the included studies.

Beyond data presentation, the collected real-time health data are used to generate personalized feedback^40,47,50,51^. For example, in ref. ^47^, collected weight data was used to trigger customized feedback, such as alert messages if the weight exceeded the standard. In ref. ^50^, the Yousician mobile application uses the device’s microphone to capture and assess the user’s musical performance, which triggers real-time auditory feedback from a keyboard with concurrent visual feedback on timing accuracy. Similarly, in ref. ^51^, both auditory and visual feedback are generated based on the collected data.

In six interventions, data are not only presented to patients but also shared with healthcare professionals to enhance monitoring, provide timely feedback, and support personalized care. For instance, the RehabPhone^51^ application employs cloud services to synchronize data across multiple devices, ensuring that both patients and healthcare providers can access and review long-term performance metrics. In ref. ^33^, the tablet application is complemented by a web portal where staff, such as community nurses and care managers, can monitor participants’ progress toward their goals. Through this portal, they can push text messages to patients’ tablets, offering informational or motivational content. Similarly, in ref. ^44^, patients can self-track their medication adherence, with data automatically sent to a website for professional analysis and communication with patients. In ref. ^46^, patient input data is uploaded to a system database, allowing doctors to remotely view the latest health records of patients. Two interventions^45,48^ link collected data with electronic health information systems. In ref. ^45^, an interactive web-based system was integrated with electronic health records (EHRs), enabling real-time monitoring and immediate response from healthcare providers. Similarly^48^, developed a mobile application that enabled physicians to view patient data in real time between clinic visits, incorporating lifelog data from personal sensors and wearables into electronic medical records.

Another two interventions^38,49^ use advanced data processing to provide more sophisticated feedback mechanisms. For example, in ref. ^38^, a statistical framework was used to provide real-time, personalized blood glucose forecasts by integrating self-monitoring data with physiological models, enabling continuous updates and more accurate predictions to assist patients in daily management tasks. Similarly, in ref. ^49^, both static data from a baseline database and dynamic behavioral data from patients were fed into a message-matching module to select appropriate messages tailored to the patient’s needs. This system also included a behavioral analysis module, which extracted information from the dynamic database to assess patient adherence and behavior, refining the feedback strategy. However, in four studies^39,42,52,53^, the collected data was used solely for recording purposes or study analysis without triggering any further actions within the intervention. For example, in^52^, the focus was more on recording patient-generated data rather than using it to trigger feedback mechanisms or alerts; in ref. ^39^, although data from pedometers was processed, it was used solely for study analysis and was not integrated into the intervention itself.

### Motivational strategies to improve patient adherence

To understand the factors contributing to increased adherence to patients’ self-care behavior, we analyzed the motivational strategies employed in each study. These strategies can be broadly categorized into seven types: feedback, reminders and motivational messages, goal setting and tracking, social interaction, health literacy, reward, and gamification. Multiple motivational strategies were often combined to enhance user engagement and adherence. Details on applying these strategies and the related theoretical underpinnings in each intervention are presented in Table 3.

**Table 3:** Motivational strategies

Strategies	Studies	Details	Theoretical models and constructs
Feedback (*n*=12)	^40^	Paired information (HF self-management skills and knowledge) and motivation (using HeartMapp as a health care coach with automated alerts and feedback) animated biofeedback deep breathing exercises and walking	IMB model
	^49^	An automatic feedback module provides personalized feedback and educational support, i.e., tailored messages based on 18 scenarios	NR
	^38^	Personalized Forecasts: providing in-the-moment meal-time decision support using personalized forecasts to predict changes in BG levels in response to meals; GlucOracle provides patients with correctness feedback and explanations based on users nutritional assessment	NR
	^51^	A feedback module uses auditory (compliments, fanfare sounds) and visual (progress graphs, scores) feedback to motivate users to understand and improve their performance	NR
	^39^	Feedback loop, e.g., motivation is enhanced as levels of PA increase	SDT
	^50^	Concurrent visual feedback on timing accuracy and key-pressing correctness	NR
	^48^	Personalized recommendations and bidirectional feedback to each participant every 2 weeks through the iCareD system	NR
	^47^	Customized feedback (i.e., clinical decision support) will be triggered if a patient’s weight exceeded heart failure monitoring standards	SST
	^41^	Immediate and cumulative feedback on adherence & BP levels	NR
	^46^	A health check-up module analyzes the patient’s BP trends and hypertension grade and returns feedback to the patient	NR
	^45^	Timely feedback about participants’ clinical variables (e.g., home BP readings, medication doses, weight, and steps)	NS
	^43^	Automated text feedback for diabetes patients	NR
Reminders and motivational messages (*n* = 11)	^42^	A self-monitoring ‘assistant’ that automatically creates motivational messages to promote optimal self-care	NR
	^40^	Tailored automated reminders are sent to patients to check their weight and complete HF symptom assessment questions and medication	
	^33^	Users can set reminders to help them stay on track with their goals, ensuring consistent engagement and progress (competence)	SDT
	^44^	Weekly motivational messages are sent to encourage adherence. Medication reminders and healthy lifestyle advice through reminder pushes	NR
	^41^	Personalized motivational and reinforcement messages based upon adherence levels; reminder signals and smartphone text messages reminded patients to measure blood pressure	SDT
	^47^	Motivational message	SST
	^52^	Behavioral trigger messages called sparks (designed for individuals who could benefit from motivational support)	SCT, FBM
	^48^	Automated motivational messages	NR
	^39^	Encouragement messages and reminder emails were sent if participants missed goal reviews or website logins, with prompts to review goals the next day	SDT
	^53^	Daily reminders	HBM
	^46^	A reminder service module provides customizable reminders for self-management tasks, allowing users to set, adjust, and close reminders based on their personal schedules	NR
Health literacy (*n* = 9)	^42^	The application provides educations that help increase the patient’s knowledge about managing his or her HF	NR
	^53^	Educational resources (short articles and short videos) on self-management of hypertension)	HBM
	^47^	Educational messages about heart failure self-management were derived from the heart failure teaching material	SST
	^52^	Each module within capABILITY consists of 3 weeks of unique educational material related to that particular core education module	SCT
	^48^	Automated educational messages	NR
	^40^	HF education (CHF-info feature) includes 10 educational modules specific for HF and common chronic diseases associated with HF. The HF-info feature in the HeartMapp includes audio-enabled interactive teaching tools on the nature of heart failure, the importance of low-salt diets, exercise regimen, HF medications, and managing other chronic diseases or conditions and feelings about HF as well as heart and brain connection	IMB model
	^46^	the health education module provided health knowledge about chronic diseases to patients in various forms	NR
	^45^	Web-based education handouts and feedback message nuggets (texts, links to Web pages, or short videos) that could be sent via secure messaging on the Web-based patient portal throughout the intervention	NS
	^49^	Educational content and quiz, for reading educational topics and answering quizzes; an automatic feedback module to provide self-management and educational support through feedback messages about physiological status, prescription recommendations and tips	NR
Goal-setting and tracking (*n* = 6)	^52^	Setting a weekly goal, acknowledgment of meeting the goal at the end of the week	SCT
	^33^	Setting SMART goals: The goal elicitation and goal setting modules use motivational interviewing to align goals with users’ needs, values, and preferences (autonomy); the goal tracking module allows users to track their goals by tracking frequencies (daily, weekly, monthly, quit).	SDT
	^48^	To encourage PA, the goal of a step count of >10,000 steps per day was set, and this goal was adjusted according to underlying diseases or individual health conditions	NR
	^39^	Setting weekly step and physical activity (SMART) goals and developing plans to achieve these goals	SDT
	^53^	Goal setting	HBM
	^45^	Setting 2 to 3 small attainable goals utilizing motivational interviewing techniques in a Web-based dashboard	NS
Social interaction (*n* = 4)	^46^	Leaderboard Module: The leaderboard module provided social support for patients and improved their compliance by indirectly improving their motivation	NR
	^43^	Mediated peer social support between patient and a patient-selected supporter (i.e., a family member or friend who is asked by the patient to serve in the support role and who agrees to do so)	NR
	^39^	Social support—encouraged to seek support from friends and family/exercise coach to implement and maintain PA	SDT
	^33^	The app enhances relatedness by sharing example goals set by other users or study participants and enabling communication with AiP staff for support	SDT
Reward (*n* = 2)	^50^	Rewards participants who collect “stars” and provides “certificates,” which are displayed on a summary report screen after finishing each exercise	NR
	^33^	Acknowledges user’s efforts by rewarding them with virtual crowns and trophies	NR
Gamification (*n* = 1)	^46^	Leaderboard Module: All the behaviors of patients concerned with self-management were converted into scores, and patients could view their real-time leaderboards to compare themselves with other users through the application	NR
		The health report module calculated scores based on the completion status of provided tasks and on changes in health data	NR

*HF* heart failure, *PA* physical activity, *BP* blood pressure, *BG* blood glucose, *IMB* information-motivation-behavioral skills model, *SDT* self-determination theory, *SCT* social cognitive theory, *HBM* health belief model, *SST* situation-specific theory, *FBM* fogg behavior model, *NR* not reported, *NS* not specified

Table 3 outlines the motivational strategies identified in the reviewed studies. The table is organized into four columns: strategies, studies, details, and theoretical models/constructs.

Twelve interventions provided personalized feedback to patients on their behavior and performance related to self-care, often also including alerts or recommendations^38–40,46–51^. For instance, in ref. ^38^, personalized blood glucose forecasts were offered to assist with meal-time decisions, which patients found reassuring and motivating, with one participant mentioning, “It would also tell me when I was doing wonderful. I am taking care of my sugar." In ref. ^46^, a health check-up module was included to analyze blood pressure trends and hypertension grades, providing tailored feedback. In ref. ^51^, the feedback module delivered auditory cues, such as compliments and fanfare sounds, and visual feedback based on performance metrics after activity completion. This approach is akin to that used in ref. ^50^, where the Yousician mobile application captured musical interactions and triggered real-time auditory feedback from a keyboard, coupled with visual feedback on timing accuracy. Additionally, in ref. ^43^, text messages were sent to reflect patients’ recent self-monitoring blood glucose frequency and glycemic control, with examples like, “You are doing a good job of monitoring. Your blood sugar levels are improving." However, this study highlighted a potential downside: motivational messages could sometimes be discouraging, particularly when feedback indicated that the intended behavior change had not been achieved.

Nine studies^40,42,45–49,52,53^ addressed patients’ health literacy in self-care interventions. Four studies^45,47–49^ utilized one-way messaging for providing education and information. Two of these studies^49^, and ^45^, combined the feedback with educational content in the messages: one^49^ provided prescription recommendations and health tips, while the other^45^ offered web-based educational content and feedback through messaging. The other two studies^47^, and ^48^, sent messages covering topics, such as diet, sleep, stress management, and medication adherence.

Additionally, four studies^42,46,52,53^ integrated educational materials within intervention applications to improve patients’ health knowledge, awareness, and self-care behaviors. For example^52^, featured modules on diet, exercise, and self-management, delivered through videos and text files. ^40^ included a “CHF-info" module with 10 educational units focused on heart failure and related conditions.

Eleven studies provided reminders and/or motivational messages to increase patient adherence^33,39–42,44,46–48,52,53^, included automated reminders^40,41,44,53^, patient-set reminders^33,39,46^, and personalized reminders arranged by external parties^39^. For instance, in ref. ^46^, a reminder service module was implemented, where the reminder times were initially set according to the management plan, with results indicating that this feature was particularly beneficial for patients with limited self-management experience, helping them complete daily tasks. Similarly, in ref. ^40^, the application sent real-time custom automated reminders to patients, prompting them to check their weight and complete HF symptom assessments. Although the intervention did not significantly enhance medication adherence, it notably increased self-care confidence compared to a control group that only received HF education.

Motivational messages were utilized in seven of these studies^39,41,42,44,47,48,52^. These messages, also referred to as reinforcement or behavior-triggered messages in some studies, were pervasive, being used to encourage adherence in various contexts^39,41,42,44,47,48^. For instance^48^, automatically sent motivational text messages alongside educational and behavioral content, while ref. ^41^ provided tailored motivational and reinforcement messages based on patients’ previous day’s medication adherence. In ref. ^39^, patients were invited to write personal motivational messages to be sent weekly or monthly, reinforcing their commitment to becoming more active. Additionally, in ref. ^52^, two types of motivational messages were used to send to participants based on the Fogg behavior model (FBM)^55^: “sparks," which motivate those needing encouragement, and “facilitators," for individuals lacking ability or capacity. The study found that spark triggers prompted specific behaviors more quickly than facilitator triggers or no triggers, although the difference was not statistically significant. However, the quicker response to spark triggers highlights their potential as a crucial element in prompting timely behavior, aligning with FBM’s emphasis on the need for motivation, ability, and a trigger to complete a task^52,55^.

Goal setting, as a commonly used motivational strategy, has been observed in six included studies^33,39,45,48,52,53^. Goal setting is often combined with planning and visual indicators for progress tracking^33,53^. In ref. ^48^, researchers set goals for patients to encourage physical activity. In contrast, in refs. ^33,39,52,53^, the goals are set by the users themselves. Participants in ref. ^39^ reported that while aiming to meet or exceed step goals was motivating, the fear of failing to meet these goals also drove motivation. Similarly, significant improvements were observed in ref. ^53^ in self-efficacy through goal setting, which likely enhanced participants’ confidence. The intervention in ref. ^33^ was specifically designed to support goal-oriented care with three modules: goal elicitation, goal setting, and goal tracking, designed to address autonomy, competence, and relatedness according to self-determination theory. Results indicated that, in practice, goal-setting and expectation management were predominantly assisted by care managers, which emphasized that digital interventions are often more effective when supplemented with human support^33,56^.

Social interaction is featured in four studies, which involved interaction among peers^33,46^ or between patients and their supporters, such as friends, family members, or healthcare providers^39,43^. For example, the application developed in^46^ employed a leaderboard feature that allowed users to compare their progress with others, thereby utilizing social support as a motivational tool. Similarly, social support was incorporated in^33^ by allowing users to view goal lists set by others within their social network. Participants found this feature informative and helpful, suggesting that integrating social network insights into digital interventions can enhance user engagement and trust^33^. In ref. ^43^, it was demonstrated that pairing adults with Type 2 diabetes with a patient-selected supporter in a mobile information and communication technology (ICT) intervention not only enhanced emotional and instrumental support, improving patients’ self-efficacy but also provided supporters with valuable insights and awareness about the challenges faced by diabetes patients, helping them more effectively reinforce positive behaviors in their paired patients. Additionally, in ref. ^39^, the program did not directly facilitate social interaction but offered resources, such as online links to local support services and encouraged users to create and share personal ’physical activity challenges’ with their social network, aiming to foster and strengthen social support networks.

In ref. ^46^, a leaderboard module was implemented in the designed mobile health application, using persuasive technology^57^ by motivating users to aim for higher scores to see their names on the leaderboards. This not only provided a competitive edge but also served as a source of social support, boosting motivation and indirectly improving compliance. Moreover, the health report module described in^46^ implemented a scoring system based on task completion and changes in health data, with the results indicating that patients who were more educated and motivated to learn about their condition found this module particularly effective in enhancing their compliance with prescribed treatments.

The reward strategy was employed in two studies^33,50^. In ref. ^33^, participants earned virtual crowns and trophies for their efforts, which they found motivating. Interestingly, the study^33^ also implemented negative rewards by using sad face icons when goals were unmet. Some participants reported that these negative rewards were also motivational; to avoid seeing a negative icon on their dashboards, they worked harder to achieve their goals^33^. However, some desired more tangible incentives, such as vouchers. In ref. ^33^, participants unlocked new levels and earned “stars" and “certificates" as rewards, displayed on a summary screen. The music-based nature of the intervention^50^ also provided an enjoyable and rewarding experience, enhancing engagement in rehabilitation.

### Theoretical foundations in motivational strategies

Not all studies specified the theoretical underpinnings for the aforementioned motivational strategies. 41% (*n* = 7) of them did not explicitly mention the application of psychological theories, models, or principles in the design or implementation of their interventions. However, many of these strategies inherently align with well-established theories employed by the other studies.

The remaining ten studies cited a total of eight different theoretical models and constructs. Self-determination theory (SDT)^58^ was referenced in three studies^33,39,41^, which posits that humans have three innate psychological needs: autonomy, competence, and relatedness. This theory primarily supported strategies related to goal setting and autonomous regulation, aiming to enhance self-efficacy and foster intrinsic motivation. Social cognitive theory (SCT)^59^ was cited in two studies^45,52^. In ref. ^52^, SCT was used to design and reinforce social modeling statements in educational videos to boost self-efficacy beliefs; one example was “others like yourself have been successful in managing their type II diabetes." This was implemented alongside the FBM^55^ to develop trigger messages to coincide with the use of the RMT. The other study^45^ integrated SCT with the theory of planned behavior^60^ and the health belief model (HBM)^61^ to enhance the effectiveness of the intervention without clearly outlining the distinct contributions of each theory. Additionally, the HBM^61^ was used in ref. ^53^ to guide strategies to enhance self-efficacy through educational resources and to use goal setting and reminders for regular task performance. Situation-specific theory (SST)^62^ was applied in ref. ^47^, where it focused on self-monitoring of symptoms, health literacy, and self-efficacy. This theory guided the strategies of educational and motivational messages within the intervention. The IMB model^27^ was used in ref. ^40^ to combine information on HF self-management with the RMT as a healthcare coach to enhance intrinsic motivation and improve engagement. The model specifies that health promotion behaviors begin with health promotion information and motivation, which subsequently lead to the development of skills required to initiate and sustain these behaviors^27^.

### Evaluation of patient adherence to self-care

Seventeen studies evaluated patient adherence to various self-care behaviors, including behavioral adherence to prescribed activities (*n* = 8), application usage/engagement (*n* = 6), and medication adherence (*n* = 3). Some studies^40,42,47–49,53^ assessed adherence to more than one activity. Sustained engagement over time is considered a dimension of adherence in this review, as it reflects consistent participation and serves as a facilitator of overall adherence^63^.

Adherence to application usage was normally assessed through the time spent on the applications^40,46,50,52^ and the frequency of usage across different features^33,38^. Medication adherence was evaluated in three studies, each employing distinct methods: one study^40^ used the self-administered Morisky medication adherence questionnaire, another^44^ utilized the proportion of days covered with medication along with the simplified medication adherence questionnaire (SMAQ), and a third study^41^ measured adherence based on the timing of medication intake relative to the prescribed dosing schedule. Nine studies focused on behavioral adherence to prescribed activities, including self-monitoring of vital signs (e.g., blood pressure, blood glucose levels, and body temperature)^42,43,45,48,49,53^ and dietary^54^, completion of educational content and quizzes^53^, and performing physical activity tasks^39,48,51^. Most of these studies measured adherence by assessing the frequency or degree to which patients followed medical prescriptions and their success in achieving set health goals. It was worth noticing that in ref. ^49^, they used a set of indexes to estimate the patient’s adherence to the treatments. Additionally, in ref. ^47^, the self-care of heart failure index (SCHFI) was used to subjectively assess adherence by evaluating participants’ self-care maintenance, management, and self-confidence.

In total, 59% of the included studies (*n*=10) reported high or increased adherence rates in their intervention with motivational strategies^33,40–42,44,46,48,49,51,53^. Among these, two RCTs showed significantly higher adherence in intervention groups compared to controls. The smartphone medication adherence stops hypertension (SMASH) intervention^41^ led to sustained medication adherence, highlighting the importance of self-efficacy and autonomous regulation. Similarly, in ref. ^48^, participants who received personalized feedback (MPC group) engaged more consistently with the app, although this engagement declined to 50% by the study’s end. Another three RCTs^40,42,49^ reported high adherence in the intervention groups, but results were not statistically significant or lacked *p*-values. In ref. ^42^, patients, including those with poor digital literacy, maintained high adherence to self-monitoring throughout the study, indicating the application’s sustainability. Two studies^40,49^ reported overall improvements in adherence, but they did not include detailed statistical information, such as *p*-values, to show whether these improvements were significant.

Two longitudinal studies reported user adherence exceeding 90%, indicating high levels of sustained engagement over time^51,53^. In ref. ^51^, patients preferred using their smartphones over traditional rehabilitation tools, though some technical issues affected adherence. In ref. ^53^, goal-setting and daily reminders in the application were found to significantly improve users’ self-efficacy and help establish effective hypertension management habits. A pre-post study^44^ showed increased adherence based on self-reports, though this was not reflected in medication coverage, suggesting the need for multiple adherence measurement methods. Additionally, over 60% of participants had a college or professional education, which correlated with higher adherence levels^44^. A formative evaluation^33^ revealed that participants who remained engaged valued rewards and experienced increased commitment to their health goals, while those who abandoned the application cited distrust, health issues, or technical difficulties as reasons for disengagement.

Other studies showed mixed results. A crossover study^52^ observed higher task adherence with behavioral trigger messages, though not statistically significant. Despite improvements in beliefs about physical activity in ref. ^39^, the results did not see increased activity levels at 12 months. Among the pre–post studies, usage remained stable over a three-week period in ref. ^50^, while^45^ provided engagement data without detailed results^45^; clinically relevant improvements in maintenance, self-management, and self-confidence were observed in ref. ^47^, though these results lacked statistical significance. In feasibility studies, one^43^ found that 20 to 30% of participants did not adhere to SMBG, and the other^38^ noted a decline in adherence overtime during the intervention.

## Discussion

Patients with chronic diseases (CVD and DM) often require long-term self-care to achieve optimal health outcomes. Digital interventions play a crucial role in supporting these self-care behaviors. This systematic review synthesized evidence from 2004 to 2024 on motivational strategies within digital health interventions designed to improve patient adherence to self-care. Despite an extensive literature search, only 17 studies met the inclusion criteria, underscoring the limited attention given to the specific integration of motivational strategies in the design of the self-care intervention and highlighting a pressing opportunity for further investigation. The following discussion analyzes the strengths and weaknesses of current approaches, including gaps in theoretical application and methodological inconsistencies, identifies key areas for improvement, and proposes possible directions for future research to optimize patient adherence and engagement with digital interventions.

All the studies included in this review utilized user control in their interventions, as we focus on self-care, where patients play an active role in managing their health. However, user control alone may not be suitable for all patient populations, particularly those with complex health conditions or lack of self-care experience where expert control becomes essential. In such cases, expert supervision can help to offer guidance, and monitor progress.

Some interventions incorporated both user and expert control, with only one study^48^ directly comparing these approaches. In ref. ^48^, the hybrid system, combining user and expert control, demonstrated better health outcomes, a greater reduction in glycated hemoglobin levels, and improved adherence, compared to the user-only control group. However, the findings from a single study cannot be generalized. Further research is needed to evaluate the effectiveness of different control approaches across diverse contexts and patient populations.

Our review examined the types of RMTs used in digital interventions, highlighting their diverse applications. Passive RMTs offer unobtrusive monitoring and continuous data collection without requiring active input from users. This feature aids in long-term adherence by reducing the burden of manual data reporting. In ref. ^39^, passive RMTs, pedometer, and the fridge magnet (equipped with recording strips) were used not only for monitoring but also as motivational tools. Active RMTs were more commonly used in the interventions reviewed. These systems can offer richer data by capturing a wider range of health indicators. Nevertheless, the need for active participation poses challenges, such as low user engagement and compliance. For example, in ref. ^46^, participants reported the routine of recording data as tedious and disengaging. This highlights the importance of integrating motivational features into active RMTs to enhance user engagement. Some interventions combined passive and active RMTs^39,40,48,49^, allowing continuous objective data collection while incorporating subjective user-reported metrics, which also requires sustained patient motivation. Moreover, integrating data from both sources can be complex, necessitating sophisticated algorithms and user-friendly interfaces to ensure the information is actionable and meaningful.

Our review was unable to establish definitive conclusions about the relative efficacy of passive, active, and hybrid systems due to the varied methodologies and adherence outcome measurements used across the included studies. Further exploration of the strengths and limitations of these systems in different contexts is needed to better understand their relative efficacy.

This review has found evidence suggesting digital interventions incorporating motivational strategies can positively influence patient adherence to self-care behaviors. However, their effectiveness often varies and is not always statistically significant due to factors like study design limitations (e.g., small sample sizes or short durations) and the context, user characteristics, and specific self-care behaviors targeted. These variations highlight the need for tailored approaches rather than one-size-fits-all solutions. To address this, we recommend adopting user-centered approaches like co-creation or participatory design, involving collaboration between patients, healthcare providers, and other stakeholders, when designing motivational strategies. This can help tailor interventions to individual needs and contexts, ultimately maximizing their impact on adherence and health outcomes.

While several studies^40–43,48,52,53^ have explored the relationship between the proposed digital interventions and patient self-efficacy, there is a notable absence of evidence supporting a clear pathway from the design of motivational strategies to improvements in self-efficacy, and ultimately, to sustained behavior change, i.e., adherence. Despite these gaps, the review showed that certain features of digital interventions regardless of the applied platform, which are feedback, health literacy, reminders and motivational messages, goal-setting, social interaction, gamification, and reward can serve as motivational strategies. These features can potentially improve self-care practices and, consequently, enhance adherence to the intervention. While we could not definitively conclude which specific feature is more effective in motivating patients, the evidence suggests that combining multiple strategies is more likely to yield positive outcomes. Further research can explore the contextual factors influencing the effectiveness of various motivational strategies and test their impact in diverse settings.

Previous reviews have found that effective behavior change interventions often include personalized, tailored, and actionable feedback^64^. Our findings are consistent with this, as personalized feedback is widely used and recognized as a key motivator in the reviewed studies, which not only provides patients with real-time reflections on their health behaviors but also serves as an emotional touchpoint, reinforcing positive behavior. However, its effectiveness depends heavily on timing and how the feedback is worded and presented; poorly delivered feedback can discourage patients^43^. To optimize this, future interventions could consider adaptive feedback systems, tailoring feedback based on user changing behavior and contextual state. For example, AI-driven systems can provide context-sensitive, supportive messages that align with individual progress and emotional states^65^. Additionally, reminders and behavior-triggered messages are often used alongside feedback. Many studies reviewed employ motivational messages, which have been found to outperform simple reminders in encouraging desired behaviors^52^. Therefore, we suggest that incorporating positive reinforcement and behavior-triggered reminders could enhance patients’ intrinsic motivation over time, potentially leading to sustained behavior change.

Goal setting has shown promise in sustaining motivation. Our review found that most studies allowed patients to set and modify their goals, which gave them control over their health journey as their needs and priorities changed^33,52^. This highlights the need for future research to develop flexible, patient-centered goal-setting frameworks that can adapt to individual progress. Additionally, evidence indicates that patients who set goals with the guidance of healthcare professionals, such as through purpose-built applications^33^, tend to manage their conditions more effectively. This underscores the importance of professional support in enhancing patients’ ability to manage their health^66^.

Enhancing patients’ health literacy has emerged as another important strategy in the reviewed studies. Interventions that incorporate educational content could increase patients’ understanding of their conditions and improve self-management skills. However, the effectiveness of these interventions can vary depending on how the content is delivered. Approaches range from one-way messaging to integrated educational modules, with evidence suggesting that the format and delivery method significantly impact the effectiveness of health literacy efforts. Moreover, fostering social interaction has proven to be an important element in enhancing adherence. Social support, whether through peer interaction or supporter engagement, contributed to motivation and adherence. The effectiveness of social network integration, as seen in refs. ^46^ and ^43^, indicates that fostering a sense of community and shared experience can be a powerful motivator. This finding suggests that digital interventions could benefit from incorporating more robust social features.

In addition to education and social support, rewards play an important role in motivating patients. Our review indicates that virtual rewards can be effective for older adults when well-designed and aligned with user preferences^33^. While in ref. ^33^, it was found that negative rewards could also serve as a motivational tool for certain individuals, this contradicts findings in ref. ^67^. These conflicting findings highlight the need for further research to better understand how different types of rewards influence motivation and consider exploring and testing the effectiveness of various rewards to determine their impact on user engagement and behavior change.

Gamification has been shown to effectively improve patient adherence in previous studies^68,69^. However, in our review, only one study utilized this strategy^46^. This limited use might be explained by findings from a review of mobile health applications, which indicated that game-like features are particularly appealing to teenagers but less effective for adults^70^. Given that the participant populations in the reviewed studies are predominantly older adults, this could explain the limited application of gamification strategies. This observation points out a potential gap in using gamification across diverse age groups. Future research can explore how to adapt gamification elements to be more effective for older adults or investigate alternative, equally engaging motivational strategies for various demographics.

Despite the diverse range of motivational strategies employed in digital interventions, there is a notable inconsistency in the explicit integration of theoretical frameworks. While some strategies are aligned with well-established theories such as SDT or SCT, many interventions do not explicitly reference these theories. However, not all successful strategies require formal theoretical foundations. For example, social interactions in interventions may not have originated from theory, but digital interventions incorporating social networking features can still benefit from a theory-driven design. Applying theory enhances our understanding of behavior, motivation, and social dynamics, ultimately improving intervention design and effectiveness.

Moreover, even in studies that claim to draw on theory, the connections between theoretical constructs and specific features or design decisions are often inadequately described. This lack of clarity makes it challenging to draw generalizable theoretical insights. Therefore, we recommend adopting a theory-driven approach in the design and development of digital interventions, as demonstrated in studies like^41,46^, and through research such as^52^, to maximize the potential usefulness of theory. Furthermore, intervention design should also provide clear explanations of how and why they applied and tested theoretical principles in their studies.

The role of data in digital interventions for patient self-care has shown to be multifaceted and influential. Contemporary digital interventions increasingly utilize real-time patient-generated health data to personalize feedback. The trend toward integrating advanced data processing techniques, such as predictive modeling and real-time analytics, represents a significant advancement. Emerging technologies like artificial intelligence (AI) can enhance this personalization by analyzing large, complex datasets to identify patterns and provide actionable insights^71,72^. AI can further predict adherence trends based on historical behavior and suggest timely adjustments to feedback mechanisms to better support self-care.

In addition to personalized feedback, data visualization can play a crucial role in digital interventions to aid patients in monitoring their progress and understanding their health status^73^. However, the lack of focus and insufficient detail in the descriptions of these visualizations indicate that health data visualization might not be a central focus in this field. This also suggests a missed opportunity to implement more effective and engaging data presentation methods, as literature has demonstrated that well-designed data visualization can enhance patient engagement and adherence to self-care. For example, systems like UbiFit Garden^67^ used dynamic visualizations to boost user engagement in physical activity. Additionally, Murnane et al.^74^ provided users with esthetically pleasing visualizations that tracked their activities and progress, resulting in increased physical activity, goal completion, and overall user engagement. Therefore, we recommend that future research and intervention design give more attention to developing and integrating advanced and engaging data visualization techniques. One example could be leveraging multi-modal generative AI like Stable Diffusion^75^, to create dynamic, personalized visualizations that adapt to user interests and behaviors.

Furthermore, the data collected through these digital interventions not only aids patient self-care but also facilitates enhanced care coordination through data-sharing between patients and healthcare providers. Systems like refs. ^45,48^ that enable real-time data sharing have shown promise in improving the responsiveness and accuracy of healthcare decisions. As digital health technologies continue to evolve, there is an increasing need for seamless integration with EHR and other healthcare systems to ensure continuity of care and optimize patient outcomes. Meanwhile, by extracting and analyzing comprehensive datasets from EHRs and RMT-collected health data, AI can maximize its capabilities to predict treatment responses, optimize therapy decisions, and personalize care plans for individual patients^76^.

Patient adherence is a critical factor in the success of digital interventions, yet it remains one of the most challenging aspects to achieve in practice. During the screening process for this review, 91 studies were excluded, 64 of which were excluded specifically because they either lacked adherence evaluations or focused on other outcomes instead. This suggests that adherence is often underemphasized or inconsistently addressed in digital health research, indicating a gap in the field. Including adherence-focused evaluations in future studies is essential, as understanding adherence behaviors is crucial to the effectiveness of digital health interventions.

Throughout the reviewed studies, variability was found in how adherence was measured, making it difficult to compare results and understand the impact of different interventions. This inconsistency may be attributed to the inherent complexity of adherence, as defined by the World Health Organization^77^ “the extent to which a person’s behavior—taking medication, following a diet, and/or executing lifestyle changes, corresponds with agreed recommendations from a health care provider."

Some studies in this review, like^44,46,49^, also highlighted the difficulty in measuring adherence, noting the absence of an accepted “gold standard" for assessment. Simple metrics, such as time spent on an application or frequency of usage, can not fully capture the complexity of adherence behaviors. While medication adherence is often the focus due to its direct correlation with clinical outcomes and is typically easier to track and maintain^78^, it does not provide a comprehensive picture of self-management. Other critical aspects, such as physical activity and dietary monitoring, are also vital but more complex to measure consistently. As a result, researchers have developed various methodologies to address this complexity. For instance, one study^49^ introduced a set of indexes to estimate adherence, acknowledging that a single metric cannot fully capture its multi-dimensional nature. This approach aligns with the broader understanding that a comprehensive assessment—integrating both objective and subjective data—is necessary, although further validation of these methods is required. Given these challenges, future research should prioritize the development and validation of a standardized set of tools for measuring adherence.

Adherence challenges extend beyond measurement and include behavioral and technological factors. Factors like the usability of technology^39,40,44^, the age and educational background of users^44,46,48,79^, and concerns about privacy^44^ can all impact adherence. For instance, difficulties in using wearable technology, as noted in ref. ^40^, stress the importance of designing interventions that are not only effective but also user-friendly and accessible. Many other individual or clinical factors, such as the severity of the illness or psychological conditions, can also influence adherence. While this review focused on the design and motivational strategies, future research should consider these factors when evaluating patient adherence and analyzing results.

In summary, our review identified a range of RMTs, motivational strategies, data presentation, and adherence evaluation methods currently in use. RMTs enable personalized health management, but their effectiveness heavily relies on sustained user engagement, which remains challenging. The findings suggest motivational strategies, including personalized feedback, health literacy enhancement, reminders and motivational messages, goal-setting, social support, gamification, and rewards, emerged as potentially impactful in fostering self-care adherence. The success of these strategies is influenced by the variability in contexts, user needs, and health goals. As such, user-centered approaches can help tailor interventions to individual needs and contexts, ultimately maximizing their impact on adherence and health outcomes. Moreover, the lack of theoretical frameworks in some interventions and the insufficient or improper use of frameworks in others weaken the ability to draw generalizable insights across studies. Adopting a theory-driven approach could enhance the design and efficacy of future digital interventions.

Adherence itself presents complex challenges, and the reviewed studies highlighted the variability in adherence definitions and measurement approaches. Many interventions rely on single, basic metrics, which often fail to capture the multi-faceted nature of adherence in chronic disease self-management. This suggests a need for standardized, multi-dimensional adherence metrics that would allow for more reliable evaluations and cross-study comparisons of digital interventions’ impacts. Furthermore, this review found that data presentation methods are underutilized, particularly those employing real-time, personalized feedback and advanced data visualization. When implemented effectively, these methods can provide patients with actionable insights and reinforce engagement in self-care behaviors. Future research should explore advanced and effective data visualization to enhance patient understanding of health progress, potentially leading to improved engagement and sustained adherence.

## Methods

This systematic review was conducted following the guidelines of the preferred reporting items for systematic reviews and meta-analyses (PRISMA)^80^. Instead of registering the review protocol in a register, we describe it in detail as follows.

### Research questions

The research questions guiding this review were formulated using the patients, intervention, comparison, outcome (PICO) format (patients, intervention, comparison, outcome)^54^. The following four research questions (RQ) were defined:

- RQ1: What types of RMT were developed to facilitate self-care for patients with CVD or DM?
- RQ2: What motivational strategies were applied to promote patient adherence to self-care?
- RQ3: How has the collected data been presented to the patients for facilitating self-care?
- RQ4: How was the patient adherence to self-care evaluated, and what were the results?

### Eligibility criteria

This review is focused on studies of digital interventions within the healthcare domain. Studies were included if they met the following criteria: (1) the study population consisted of patients of any age diagnosed with any type of CVD or DM; (2) it provided a detailed description of the design or implementation of a digital intervention for promoting self-care; (3) the digital intervention involved the collection and usage of health-related data through RMT(s); (4) the study included one or more motivational strategies to prompt patient adherence; (5) the study included an evaluation of the patient adherence or sustained engagement to self-care behavior; (6) the study was published in peer-reviewed journals or conference and available in English; (7) the study was published in the recent 20 years, from the year 2004 onward, a period characterized by a shift toward more advanced, consumer-centered RMTs^81^.

Studies were excluded if: (1) the data collected by the RMTs was used solely by third parties (such as caregivers, family members, or health professionals) rather than by the patients themselves; (2) the study involved patients diagnosed with any mental disease, as these often require specialized intervention strategies that are beyond the scope of this review; (3) the study involved other factors influencing patient participation in self-care (e.g., morbidity following cardiac events or the severity of the disease); or (4) the publication was a conference presentation, poster, book review, or editorial.

### Search strategy

A literature search was conducted in four databases: PubMed, IEEE Xplore, ACM, and Scopus. The determination of search terms was an iterative process, beginning with trial searches based on an initial set of known, relevant articles, followed by multiple rounds of testing and refining until the final search query reliably identified all articles in the initial set. The search strategy included keywords and medical subject headings (MeSH) terms, along with their synonyms and spelling variations, applied to the fields of title and/or abstract. It comprised four clusters of search terms representing the study population, self-care, digital interventions, and adherence, which were employed in various combinations to tailor to different databases. Details are listed in Table 4.

**Table 4:** Literature search strategy

Population	(patient or patients) and chronic and ("heart diseases" or “vascular diseases" or “heart disease" or “heart diseases" or cardiac or “cardiovascular diseases" or “cardiovascular disease" or “coronary artery disease" or “coronary artery diseases" or “heart failure" or hypertens* or “high blood pressure" or stroke or “heart attack" or “diabetes Mellitus" OR diabetes*)
	and
Intervention	((mobile or wearable or sensor or data or digital or remote) and (health or healthcare)) or (mHealth or eHealth)
	and
Outcome	Self-management or “self management" or “self monitoring" or self-monitoring or self-awareness or “self awareness" or self-reflection or “self reflection" or self-improvement or “self improvement" or self-care or “self care" or self-report or “self report" or self-logging or “self logging" or self-tracking or “self tracking*" or “quantified self" or quantified-self or “self quantif*" or “self-quantif*" or feedback
	and
User behavior and perception	Adherence or compliance or engagement or sustain*

Table 4 outlines the literature search strategy used in this systematic review. The strategy is structured in population, intervention, outcome, and user behavior/perception. It combines search terms with Boolean operators

### Study selection

The search was conducted by the first author (TQ) on the third of July, 2024. All search results were imported and merged into EndNote 21 Referencing Software, and duplicates were removed. Titles and abstracts were then screened independently by the two review authors (TQ and QY) to eliminate irrelevant articles. Articles identified for full-text review were assessed against the eligibility criteria by TQ and QY, independently. In cases of disagreement, TQ and QY discussed the discrepancies; if a consensus had not been reached, the third reviewer (B) would have been taken into consideration. Reasons for the exclusion of studies were recorded.

### Data extraction and synthesis

A data extraction template, following the PRISMA checklist, was iteratively developed by all four authors and piloted on 2 full-text articles. Two review authors (TQ and QY) independently extracted data using this template and synthesized the main results of the included studies in a narrative manner focusing on the following perspectives: (1) general study information (author, year of publication); (2) RMTs (technology used, data collection); (3) intervention; (4) motivational strategies (designed system/feature(s), applied theory); (5) evaluation (study population, study design, adherence measurement, outcomes on adherence). For studies involving an iterative or design process, only study data from the final stage evaluation has been extracted. The screening process was conducted without blinding the study authors, institutions, or journals.

## Supplementary information

Supplementary Information

## Data Availability

Data are provided within the manuscript or supplementary information files.
